# Dilutional acidosis during whole lung lavage under general anesthesia due to excessive absorption of normal saline

**DOI:** 10.1186/s40981-022-00520-9

**Published:** 2022-04-14

**Authors:** Mikako Inada, Izumi Kawagoe, Osamu Kudoh, Daizoh Satoh, Chieko Mitaka, Masakazu Hayashida

**Affiliations:** Department of Anesthesiology and Pain Medicine, Juntendo University School of Medicine, 2-1-1 Hongo, Bunkyo-Ku, Tokyo, 113-8421 Japan

**Keywords:** Dilutional acidosis, Pulmonary alveolar proteinosis, Whole lung lavage

## Abstract

**Background:**

Whole lung lavage (WLL) is an effective therapy for pulmonary alveolar proteinosis. We report a rare dilutional acidosis following WLL in a female patient.

**Case presentation:**

Under general anesthesia, a left-sided double-lumen tube was inserted with its bronchial lumen connected to the saline delivery system. Preoperatively, arterial blood gases were within normal limits. During 14 l of fluid was instilled into the lung for 2.5 hours, a decrease in pH, K^+^, and base excess, alongside an increase in Na^+^ and Cl^−^, indicated a strong ion difference; the diagnosis was dilutional hyperchloremic metabolic acidosis. Although she remained hemodynamically stable and had no indicators of massive absorption, she stayed in the ICU for mechanical ventilation for one night out of concern of pulmonary edema.

**Conclusions:**

Inappropriate irrigating fluid pressure might lead to absorption of normal saline. Continuous monitoring and careful observation during WLL can help prevent intraoperative dilutional acidosis.

## Background

Pulmonary alveolar proteinosis (PAP) is a rare disorder in which lipoproteinaceous material accumulates within alveoli. The most effective therapy for PAP is whole lung lavage (WLL) [1]. During WLL, repeated lavage washes out the proteinaceous material from the alveoli and reestablishes effective oxygenation and ventilation. The procedure can be complicated by hemodynamic and oxygenation fluctuation. We report here dilutional acidosis in a patient with PAP during WLL under general anesthesia. The institutional review board of our hospital approved this case presentation, and the patient gave written informed consent for the publication of this case report and all accompanying images.

## Case report

A 37-year-old female, 159 cm tall, weighing 53 kg, presented with progressive exertional dyspnea for 6 months. A diagnosis of autoimmune PAP was confirmed based on her history, high-resolution computerized tomography (HRCT), and bronchoalveolar lavage (BAL) findings. WLL of the left lung, in which pulmonary infiltrates were denser than in the right lung on HRCT, was performed under general anesthesia due to exacerbation of dyspnea during follow-up. The first lavages were performed with 10,000 ml of normal saline, with an almost equal volume of returning effluent. Although her postoperative course was smooth for 4 months, she again developed exertional dyspnea and new and denser pulmonary infiltrates. Thus, a second WLL was planned 8 months after the first WLL.

Arterial blood gas (ABG) values before the second WLL, with the patient breathing room air, are as shown in Table 1. Pulmonary function tests revealed a restrictive pattern: vital capacity (VC)=1.44 (44.1%), forced expiratory volume in 1 s (FEV_1_) =1.07 (39.8%), and carbon monoxide diffusion capacity (DLCO)=4.80 (20.8%). A 6-min walk test showed desaturation with exercise from 91 to 73%, resulting in the test being aborted after 3 min. Since the radiological involvement was greater on the left side, a repeated left lung lavage was planned with stand-by extracorporeal membrane oxygenation (ECMO) to prevent fatal hypoxemia.

**Table 1:** Arterial blood gas analysis before, during, and after lavage of the left lung

	Spontanous ventilation under room air	After degassing	After 12th lavage	After completion of lavages	10 hours after lavage in ICU
		(FiO_2_= 1.0, BLV)	(FiO_2_=1.0, OLV)	(FiO_2_= 1.0, BLV)	(FiO2 = 0.4 BLV)
pH	7.425	7.387	7.349	7.337	7.366
PaO2 (mmHg)	57.5	94.9	249.7	326.8	188
PaCO2 (mmHg)	36.3	41.9	26.3	36.8	39.3
Na (mEq/l)	139.8	136.4	138	138.8	136.5
Cl (mEq/l)	105	104	116	113	109
HCO3- (mEq/l)	23.3	24.6	14.2	19.3	22
K (mEq/l)	3.87	3.41	2.77	3.41	3.95
SID (mEq/l)	38.67	35.81	24.77	29.21	31.45
base excess (mEq/l)	-0.7	-0.4	-10.2	-5.9	-2.6
Hb (g/dl)	12.9	11.6	8.8	9.9	11.6
glucose (mg/dl)	97	201	50	87	141

*ABG* arterial blood gas examination, *BLV* bilateral ventailation, *OLV* one lung ventilation

After entering the operating room, electrocardiography, pulse oximeter (SpO_2_), and non-invasive blood pressure monitors were attached. After pre-oxygenation with 5 L/min of 100% O_2_ for 5 min, general anesthesia was induced and maintained with propofol, remifentanil, and rocuronium, and a 37 Fr left-sided double-lumen tube (DLT) was inserted. Correct positioning of the DLT was confirmed using bronchoscopy. Radial artery cannulation was performed for ABG analysis, which revealed a PaO_2_ of 467.4 mmHg following 5 min of bilateral mechanical ventilation with an FiO_2_ of 1.0. End-tidal PaCO_2_, arterial blood pressure, and bladder temperature were also monitored intraoperatively. In addition to usual monitors, a FloTrac^TM^ monitoring system (Edwards Lifesciences, California, USA) and transesophageal echocardiography (TEE) were prepared. After induction of anesthesia, baseline ABG revealed within the normal limits (Table 1). The patient was placed in the supine position with the right lung side slightly tilted downward.

The bronchial lumen of the DLT in the left main bronchus was connected to the saline delivery system. During one lung ventilation (OLV) of the right lung, after letting the patient’s left lung degas for 15 min and recruitment maneuver, ABG showed a PaO_2_ of 194.9 mmHg under an FiO_2_ of 1.0. Confirming adequate oxygenation during OLV, we started lavage. The ventilator settings were kept unchanged during OLV [PCV Peak 15 cmH_2_O, PEEP 6 cmH_2_O, I: E 1:1.5, RR 14/min]. Lavage was performed by repeatedly filling the left lung with irrigating solution while performing OLV of the right lung with an FiO_2_ of 1.0.

**Fig. 1:**
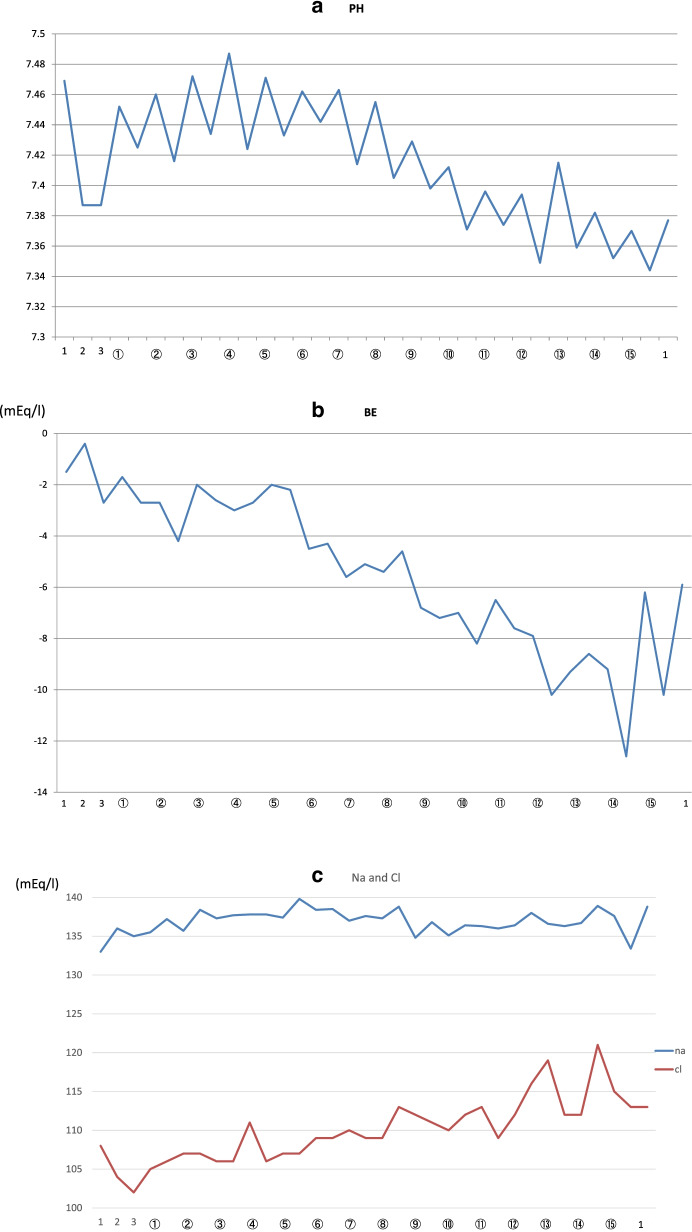
Changes in pH (**a**), base excess (BE) (**b**), and sodium (Na^+^) and chloride (Cl^−^) (**c**) during whole lung lavage (WLL). The changes show that the patient gradually developed dilutional hyperchloremic metabolic acidosis. The horizontal axis shows the time. The numbers along the *x*-axis indicate the following: 1. Both lungs ventilated at an FiO_2_ of 1.0. 2. After degassing for 15 min. 3. The beginning of lavage. White circled numbers: following saline instillation. Between the white circled numbers: following effluent drainage

In every lavage procedure, 600 to 1000 ml of normal saline flowed into the left lung at a rate of 100 ml/min from a height of 30 cm above the patient, followed by passive drainage under gravity. The procedure was repeated 15 times using the instillation of warm saline and removal of the effluent. A total of 14 l of fluid was instilled into the left lung. WLL was performed satisfactorily, with the amount of effluent removed being almost equal to the instilled volume. The effluent contained very large amounts of amorphous sediment which gradually cleared. After 2.5 h of lavage, that is, nearly at the end of WLL, ABG values are as shown in Table 1. The pH, base excess, glucose, Na^+^, K^+^, and Cl^−^ values suggested a strong ion difference (SID=20.36) (Fig. 1). Dilutional hyperchloremic metabolic acidosis was diagnosed, likely due to excessive alveolar absorption of normal saline during WLL. The intraoperative infusion was 1260 ml including 700 ml of acetate Ringer’s, 50ml of Carbonate Ringer’s, and 510 ml of normal saline. Intraoperative urine volume was 90ml.

The patient remained hemodynamically stable during WLL, and there were no significant findings suggesting massive absorption of the lavage fluid by FloTrac Sensor, TEE, or no pulmonary edema on the chest X-ray.

Due to concern of continued postoperative fluid shifts, we decided to keep the patient intubated, and the DLT was replaced with a single lumen endotracheal tube. The patient was transferred to the ICU overnight for mechanical ventilation with a positive end-expiratory pressure of 10 cm H_2_O. Additionally, furosemide was given to remove excess fluid. ABG values returned to their normal limits 10 h after WLL was completed (Table 1). She was extubated 15 h following the completion of WLL. She had no further metabolic acidosis and was subsequently discharged 4 days post-procedure.

## Discussion

There are three different types of PAP: congenital (2%), secondary (less than 10%), and acquired or adult-type PAP (90%), which is also referred to as primary or idiopathic PAP. PAP develops due to the presence of auto-antibodies against pulmonary granulocyte-macrophage colony-stimulating factor (GM-CSF), resulting in alveolar macrophage dysfunction, disruption of surfactant homeostasis, and reduced surfactant clearance from alveoli. WLL has been the standard first-line therapy for PAP since the 1960s [2].

For lavage, the normal saline instillation volume is calculated as 45% of the patient’s functional residual capacity plus tidal volume, to provide a safety margin. Maintaining the normal saline volume within this amount and keeping the instillation speed at 100 ml/min helps avoid excessive absorption of the normal saline. Since there were no significant abnormalities in ABG during the first WLL, we used the same protocol for the second procedure, but with very different results, with the gradual development of acidosis.

In the context of perioperative fluid administration, dilutional acidosis is a form of metabolic acidosis that results from rapid administration of fluids containing near-physiologic concentrations of sodium and anions (usually chloride) other than bicarbonate or bicarbonate precursors, such as lactate. The magnitude of acidosis depends on several factors: the baseline volume and composition of plasma and extracellular fluid (ECF); the volume, rate, and composition of administered fluids; the volume, rate, and composition of fluid loss; and physiological changes in ECF composition [3].

In our case, normal saline infusion caused elevation of both sodium and chloride, although chlorides increased to a greater extent, resulting in a net SID reduction and acidosis. Trans-urethral resection of the prostate (TURP) is another procedure that can cause dilutional acidosis. Although different irrigation fluids are used in WLL and TURP, absorption of distilled water during TURP can cause a decrease in sodium, potassium, and chloride. A decrease in SID leads to dilutional acidosis and hyponatremia. During TURP, the irrigation fluid either gains direct intravascular access through the prostatic venous plexus or is more slowly absorbed from the retroperitoneal and perivesical spaces. The amount of absorption is governed mainly by three factors: the hydrostatic pressure of the irrigating solution, the number and size of opened venous sinuses, and the duration of exposure [4]. In TURP, a resection time of under 1 h reportedly limits the volume of fluid absorbed by reducing the time for which the prostatic sinuses are open. Massive absorption is more likely if intravesical pressure is > 30 mmHg. Limiting the height of the irrigation bag to 40 cm above the prostate can minimize absorption [5].

Without venous or tissue damage in the WLL procedure, the absorption of saline from the lavaged lung at a rate of approximately 350 ml/h is associated with a lowering of the PaO2, an increase in venous admixture, and a lower airway pressure [6].

In general, large-volume (> 2 L) saline infusion induces hyperchloremia, resulting in metabolic acidosis [7]. Although the procedural duration of 2.5 h was within the safety margin in our case, the irrigating solution pressure was a little higher, which possibly led to excessive absorption. The amount of effluent removed being almost equal to the instilled volume. However, the removal was measured by using a plastic bag with a rough scale; it may have been overestimated in each lavage resulting in ignored absorption. Furthermore, absorbability of the irrigation fluid through the alveolar surface probably increased following the removal of the proteinaceous material. Additionally, although the volume of saline instilled in the lung and the effluent volume at each lavage cycle were almost equal, the measured effluent included an unknown amount of proteinaceous material. Hence, monitoring the extravascular lung water index might be an option for preventing volume overload when managing the irrigating fluid used for WLL.

## Conclusion

We experienced dilutional acidosis following WLL in a PAP patient. Inappropriate irrigation fluid pressure might have led to excessive absorption of normal saline. The patient recovered following overnight mechanical ventilation and diuretic therapy. Although dilutional acidosis is rare, appropriate management of the procedure, continuous monitoring, and careful observation can help prevent intraoperative dilutional acidosis during WLL.

## Data Availability

The datasets related to this report are available from the corresponding author on reasonable request.

## Abbreviations

ABG: Arterial blood gas examination
BAL: Broncho-alveolar lavage
DLCO: Diffusing capacity of the lung for carbon monoxide
DLT: Double-lumen endobronchial tube
ECF: Extracellular fluid
ECMO: Extracorporeal membrane oxygenation
FEV1: Forced expiratory volume in 1 s
GM-CSF: Granulocyte-macrophage colony-stimulating factor
HRCT: High-resolution computerized tomography
OLV: One lung ventilation
PAP: Pulmonary alveolar proteinosis
SID: Strong ion difference
SpO_2_: Saturation of percutaneous oxygen
TEE: Transesophageal echocardiography
TURP: Trans-urethral resection of the prostate
VC: Vital capacity
WLL: Whole lung lavage
